# Assessment of the Concentrations of Various Advanced Glycation End-Products in Beverages and Foods That Are Commonly Consumed in Japan

**DOI:** 10.1371/journal.pone.0118652

**Published:** 2015-03-02

**Authors:** Masayoshi Takeuchi, Jun-ichi Takino, Satomi Furuno, Hikari Shirai, Mihoko Kawakami, Michiru Muramatsu, Yuka Kobayashi, Sho-ichi Yamagishi

**Affiliations:** 1 Department of Advanced Medicine, Medical Research Institute, Kanazawa Medical University, Uchinada-machi, Ishikawa, Japan; 2 Laboratory of Biochemistry, Faculty of Pharmaceutical Sciences, Hiroshima International University, Kure, Hiroshima, Japan; 3 Department of Pathophysiological Science, Faculty of Pharmaceutical Science, Hokuriku University, Kanazawa, Ishikawa, Japan; 4 Department of Pathophysiology and Therapeutics of Diabetic Vascular Complications, Kurume University School of Medicine, Kurume, Fukuoka, Japan; University of Miami, UNITED STATES

## Abstract

Dietary consumption has recently been identified as a major environmental source of pro-inflammatory advanced glycation end-products (AGEs) in humans. It is disputed whether dietary AGEs represent a risk to human health. N*^ε^*-(carboxymethyl)lysine (CML), a representative AGE compound found in food, has been suggested to make a significant contribution to circulating CML levels. However, recent studies have found that the dietary intake of AGEs is not associated with plasma CML concentrations. We have shown that the serum levels of glyceraldehyde-derived AGEs (Glycer-AGEs), but not hemoglobin A1c, glucose-derived AGEs (Glu-AGEs), or CML, could be used as biomarkers for predicting the progression of atherosclerosis and future cardiovascular events. We also detected the production/accumulation of Glycer-AGEs in normal rats administered Glu-AGE-rich beverages. Therefore, we assessed the concentrations of various AGEs in a total of 1,650 beverages and foods that are commonly consumed in Japan. The concentrations of four kinds of AGEs (Glu-AGEs, fructose-derived AGEs (Fru-AGEs), CML, and Glycer-AGEs) were measured with competitive enzyme-linked immunosorbent assays involving immunoaffinity-purified specific antibodies. The results of the latter assays indicated that Glu-AGEs and Fru-AGEs (especially Glu-AGEs), but not CML or Glycer-AGEs, are present at appreciable levels in beverages and foods that are commonly consumed by Japanese. Glu-AGEs, Fru-AGEs, CML, and Glycer-AGEs exhibited concentrations of ≥85%, 2–12%, <3%, and trace amounts in the examined beverages and ≥82%, 5–15%, <3%, and trace amounts in the tested foods, respectively. The results of the present study indicate that some lactic acid bacteria beverages, carbonated drinks, sugar-sweetened fruit drinks, sports drinks, mixed fruit juices, confectionery (snacks), dried fruits, cakes, cereals, and prepared foods contain markedly higher Glu-AGE levels than other classes of beverages and foods. We provide useful data on the concentrations of various AGEs, especially Glu-AGEs, in commonly consumed beverages and foods.

## Introduction

In humans, two major sources of advanced glycation end-products (AGEs) have been identified, exogenous and endogenous AGEs [1–5]. AGEs are formed by the Maillard reaction, a non-enzymatic reaction between the aldehyde or ketone groups of reducing sugars, such as glucose, fructose, and glyceraldehyde, and the terminal α-amino groups or ε-amino groups of protein lysine residues [2–5]. AGEs were originally characterized by their yellow-brown fluorescent color and their ability to form cross-links with and between amino groups; however, the term is now used for a broad range of advanced products of the glycation process, including *N* ^*ε*^-(carboxymethyl)lysine (CML), *N* ^*ε*^-(carboxyethyl)lysine (CEL), and pyrraline, which are colorless, do not fluoresce, and do not form cross-links with proteins [1–5]. The use of CML as a marker of AGE formation in food has recently led to the development of a database containing the CML concentrations of 549 foodstuffs [6,7]. However, the inconsistencies between the information in this database and data obtained with other methods highlight the considerable challenges associated with analyzing AGEs [8]. Moreover, dietary CML might pose a risk to human health, as it enhances oxidative stress and initiates inflammatory responses, which ultimately lead to atherosclerosis [9,10]. While previous studies have suggested that dietary CML makes a significant contribution to *in vivo* CML concentrations [10], two recent studies have reported that this is not true for humans [11] or rats [12]. Semba *et al*. suggested that the excessive consumption of foods considered to be high in AGEs might not have a major effect on serum CML concentrations [11].

**Fig 1:**
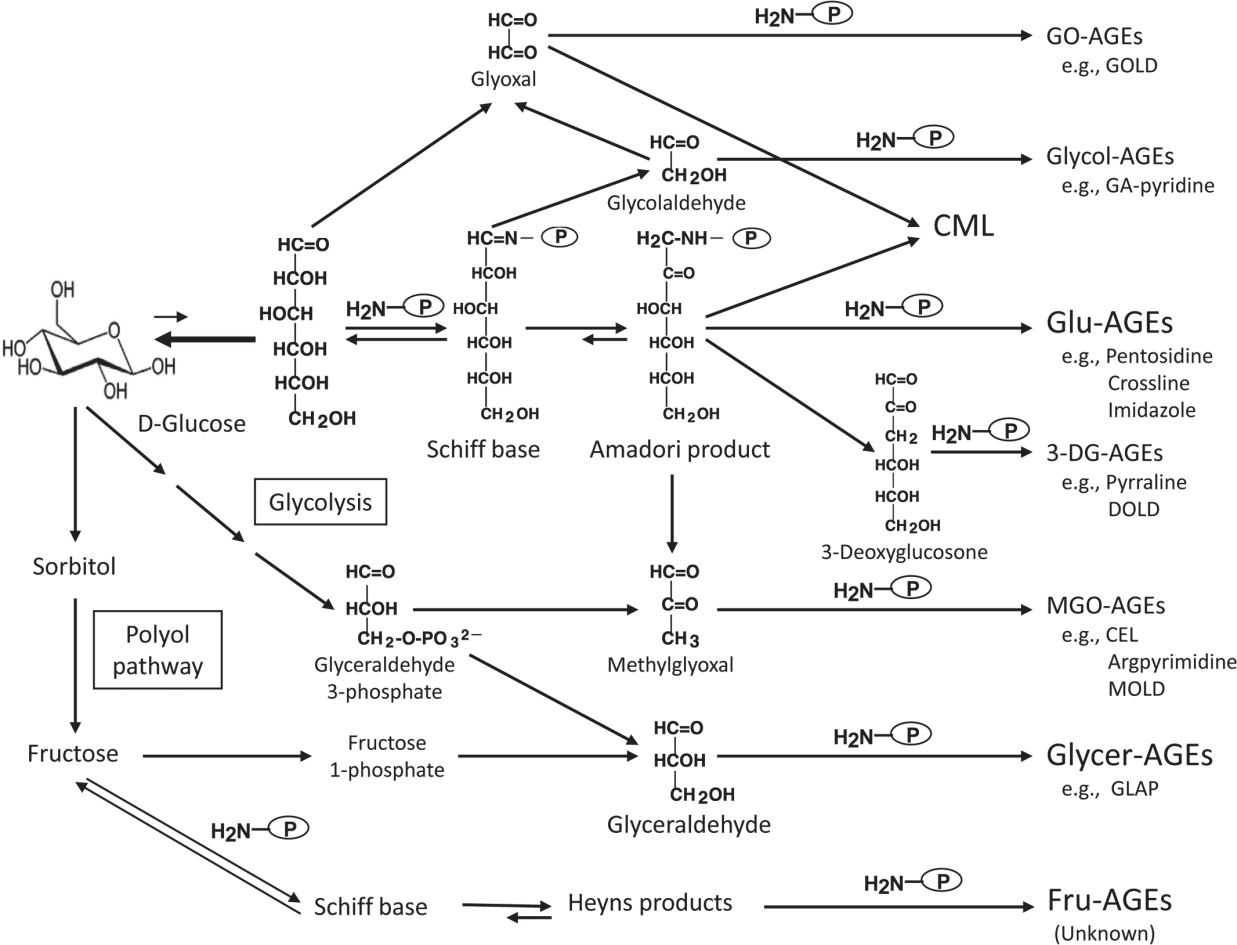
Alternative *in vivo* AGE synthesis routes. Reducing sugars, such as glucose, fructose, and glyceraldehyde, are known to react non-enzymatically with the amino groups of proteins to form reversible Schiff bases and Amadori/Heyns products. These early glycation products undergo further complex reactions such as rearrangement, dehydration, and condensation to become irreversibly cross-linked heterogeneous fluorescent derivatives, termed AGEs. Glu-AGEs, glucose-derived AGEs; Fru-AGEs, fructose-derived AGEs; Glycer-AGEs, glyceraldehyde-derived AGEs; Glycol-AGEs, glycolaldehyde-derived AGEs; MGO-AGEs, methylglyoxal-derived AGEs; GO-AGEs, glyoxal-derived AGEs; 3-DG-AGEs, 3-deoxyglucosone-derived AGEs; CML, *N* ^*ε*^-(carboxymethyl)lysine; P-NH_2_, free amino residue of protein; GOLD, GO-lysine dimer; GA-pyridine, 3-hydroxy-4-hydroxymethyl-1-(5-carboxypentyl) pyridinium cation; DOLD, 3-DG-lysine dimer; CEL, *N* ^*ε*^-(carboxyethyl)lysine; MOLD, MGO-lysine dimer; GLAP, glyceraldehyde-derived pyridinium compound.

We previously demonstrated that glucose, fructose, α-hydroxyaldehydes (glyceraldehyde and glycolaldehyde), and dicarbonyl compounds (glyoxal (GO), methylglyoxal (MGO), and 3-deoxyglucosone (3-DG)) were actively involved in protein glycation [13–16]. Seven immunochemically distinct classes of AGEs (glucose-derived AGEs, Glu-AGEs; fructose-derived AGEs, Fru-AGEs; glyceraldehyde-derived AGEs, Glycer-AGEs; glycolaldehyde-derived AGEs, Glycol-AGEs; GO-derived AGEs, GO-AGEs; MGO-derived AGEs, MGO-AGEs; and 3-DG-derived AGEs, 3-DG-AGEs) have been detected in the sera of type 2 diabetic patients on hemodialysis [13–16]. Based on these findings, we proposed an *in vivo* pathway for the formation of distinct AGEs involving the Maillard reaction, sugar autoxidation, and sugar metabolic pathways, as shown in Fig. 1.

We recently demonstrated that interactions between Glycer-AGEs and the receptor for AGEs (RAGE) affect intracellular signaling, gene expression, and the release of pro-inflammatory molecules and also induce oxidative stress in numerous types of cells, all of which can contribute to the pathological changes observed in various chronic diseases [17–19]. Furthermore, we detected increased hepatic RAGE expression and the enhanced production/accumulation of Glycer-AGEs in normal rats administered Glu-AGE-rich beverages [20]. These findings indicate that Glu-AGEs, which are frequently found in beverages and foods, and hence, are taken into the body orally, enhance the production/accumulation of Glycer-AGEs, leading to Glycer-AGE-RAGE interactions. Our recent studies indicated that the serum levels of Glycer-AGEs, but not hemoglobin A1c (HbA1c), Glu-AGEs, or CML, could be used as biomarkers for predicting the progression of lifestyle-related diseases [21–25]. We previously demonstrated that the enhanced production/accumulation of Glycer-AGEs after the oral consumption of Glu-AGEs plays an important role in the pathogenesis of vascular damage [26].

Therefore, the objective of the present study was to assess the concentrations of four kinds of AGEs; i.e., Glu-AGEs, Fru-AGEs, CML, and Glycer-AGEs, which have been detected in the sera of both non-diabetic and diabetic subjects [13–16,21–26], in beverages and foods that are commonly consumed in Japan.

## Materials and Methods

### Preparation of various advanced glycation end-products (AGEs)

Glu-AGEs, Fru-AGEs, Glycer-AGEs, and CML proteins were prepared as described in previous studies [13–16]. Briefly, 25 mg/ml rabbit serum albumin (RSA, A0764, Sigma-Aldrich, St Louis, MO, USA) or bovine serum albumin (BSA, A0281, Sigma-Aldrich) were incubated under sterile conditions with 0.5 M D-glucose, D-fructose, or 0.1 M DL-glyceraldehyde and 5 mM diethylenetriaminepentaacetic acid (Dojindo Laboratories, Kumamoto, Japan) in 0.2 M phosphate buffer (pH 7.4) at 37°C for 8 weeks (or 7 days for the samples incubated with glyceraldehyde). Low molecular weight (LMW) reactants and the glucose, fructose, or glyceraldehyde were removed using a PD-10 chromatography column (GE Healthcare UK, Buckinghamshire, UK) and dialysis against phosphate buffered saline (PBS). CML-RSA, CML-BSA, CML-human serum albumin (HSA), CML-hemoglobin (Hb), and CML-ribonuclease A (RNase A) were prepared as described previously [13]. Briefly, 50 mg/ml each of RSA, BSA, HSA, Hb, and RNase A were incubated at 37°C for 24 h with 50 mM glyoxylic acid and 150 mM sodium cyanoborohydride in 0.2 M phosphate buffer (pH 7.4), before being subjected to PD-10 column chromatography and dialysis against PBS. Protein concentrations were determined with the DC protein assay reagent (Bio-Rad Laboratories, Richmond, CA, USA) using BSA as a standard.

### Preparation of AGE-specific antibodies

Four kinds of immunoaffinity-purified antibodies were prepared as described in previous studies [13–16]. Briefly, 4 mg of three types of AGE-RSA (incubated with glucose, fructose, or glyceraldehyde) were emulsified in 50% Freund’s complete adjuvant (Wako Pure Chemical Industries, Osaka, Japan) and then injected intradermally into Japanese white rabbits (Sankyo Labo Service Corporation, Tokyo, Japan). This procedure was repeated at weekly intervals for 6 weeks. After a 2-week break, the rabbits were given a booster injection of 4 mg of the appropriate antigen. The animals were bled on the 10^th^ day after the last injection, and their sera were obtained for further affinity purification. CNBr-activated Sepharose 4B gels (GE Healthcare UK) were coupled to Glu-AGE-BSA, Fru-AGE-BSA, Glycer-AGE-BSA, or CML-BSA, as described previously [13–16]. The anti-Glu-AGE antiserum, which contained anti-Glu-AGE and anti-CML antibodies, was applied to a column (2.5 x 5.5 cm) containing Sepharose 4B coupled to Glu-AGE-BSA. After extensive washing with PBS, the adsorbed fractions were eluted with 20 mM sodium phosphate buffer containing 1 M potassium thiocyanate (pH 7.4) (elution buffer). The eluted fractions were pooled, concentrated using Centriprep-10 (Millipore Corporation, Billerica, MA, USA), and passed through a PD-10 column equilibrated with PBS. The eluted fraction was then loaded onto a column (1.5 x 5.5 cm) containing Sepharose 4B coupled with CML-BSA, which was washed with PBS to obtain the unadsorbed fraction (anti-Glu-AGE antibody). The adsorbed fraction (anti-CML antibody) was then eluted with elution buffer. The anti-Glu-AGE and anti-CML antibodies were pooled (separately), concentrated with Centriprep-10, and passed through a PD-10 column equilibrated with PBS, before being used in this study [13,15]. The anti-Fru-AGE or anti-Glycer-AGE antisera were applied to columns containing Sepharose 4B coupled to Fru-AGE- or Glycer-AGE-BSA. After extensive washing with PBS, the adsorbed fractions were eluted with elution buffer. Each of the eluted fractions was pooled, concentrated using Centriprep-10, and passed through a PD-10 column equilibrated with PBS. Then, they were loaded onto a column containing Sepharose 4B coupled to CML-BSA, which was washed with PBS to obtain the unadsorbed fraction (anti-Fru-AGE or anti-Glycer-AGE antibodies). The anti-Fru-AGE and anti-Glycer-AGE antibodies were pooled (separately), concentrated with Centriprep-10, and passed through a PD-10 column equilibrated with PBS and then used in this study [14,16].

The immunoaffinity-purified anti-Glu-AGE antibody did not recognize well-characterized AGE structures, such as CML, CEL, pyrraline, pentosidine, argpyrimidine, imidazolone, GO-lysine dimers (GOLD), and MGO-lysine dimers (MOLD). In addition, it did not recognize AGEs whose structures remain unknown, such as Fru-AGEs, Glycer-AGEs, Glycol-AGEs, GO-AGEs, MGO-AGEs, and 3-DG-AGEs [13,15,27]. Instead, the anti-Glu-AGE antibody specifically recognized unique unknown Glu-AGE structures. The immunoaffinity-purified anti-Fru-AGE antibody did not recognize well-characterized AGE structures, such as CML, CEL, pyrraline, pentosidine, and argpyrimidine. In addition, it did not recognize AGE whose structures remain unknown, such as Glu-AGEs, Glycer-AGEs, Glycol-AGEs, GO-AGEs, MGO-AGEs, and 3-DG-AGEs [16]. Instead, the anti-Fru-AGE antibody specifically recognized unique unknown Fru-AGE structures. The immunoaffinity-purified anti-Glycer-AGE antibody did not recognize well-characterized AGE structures, such as CML, CEL, pyrraline, pentosidine, argpyrimidine, imidazolone, GOLD, MOLD, and glyceraldehyde-derived pyridinium (GLAP). Furthermore, it did not recognize AGE whose structures are unknown, such as Glu-AGEs, Fru-AGEs, Glycol-AGEs, GO-AGEs, MGO-AGEs, and 3-DG-AGEs [14,23]. Instead, the anti-Glycer-AGE antibody specifically recognized unique unknown Glycer-AGE structures. The three types of AGE antibodies were able to detect both high-molecular weight (HMW) and LMW Glu-AGEs, Fru-AGEs, or Glycer-AGEs with unique unknown structures in serum [13–16,21–26]. On the other hand, the immunoaffinity-purified anti-CML antibody recognized a common epitope that is shared by protein-bound CML (such as CML-RSA, CML-BSA, CML-HSA, CML-Hb, and CML-RNase A) and free CML molecules, whereas it did not react with lysine, Amadori products (glycated HSA), unmodified proteins, pentosidine, pyrraline, argpyrimidine, imidazolone, or CEL [13]. The anti-CML antibody was able to detect both HMW and LMW CML structures in serum [13].

### Competitive enzyme-linked immunosorbent assay (ELISA)

The concentrations of four kinds of AGEs (Glu-AGEs, Fru-AGEs, CML, and Glycer-AGEs) were measured with competitive ELISA using the immunoaffinity-purified antibodies described above. Briefly, a 96-well (flat bottomed without a lid, high binding) enzyme immunoassay /radioimmunoassay plate (Corning Incorporated, Corning, NY, USA) was coated with 1 μg/ml of Glu-AGE-BSA, Fru-AGE-BSA, CML-BSA, or Glycer-AGE-BSA standard solution and incubated overnight at 4°C. The wells were washed three times with 0.3 ml of the washing solution (PBS containing 0.05% Tween-20), before being blocked *via* incubation for 1 h with 0.2 ml of a PBS solution containing 1% BSA. After the wells had been washed with the washing solution, test samples (50 μl) were added to each well as competitors for 50 μl of immunoaffinity-purified anti-Glu-AGE, anti-Fru-AGE, anti-CML, or anti-Glycer-AGE antibodies (1:1000~1:2500 dilution), and then the plates were incubated for 2 h at room temperature under gentle shaking in a horizontal rotary shaker (EYELA, MMS-1, Tokyo, Japan). The wells were then washed with washing solution and developed with an alkaline phosphatase-conjugated sheep anti-rabbit IgG (Millipore Corporation, Billerica, MA, USA) using p-nitrophenyl phosphate as the colorimetric substrate (Pierce, Rockford, IL, USA). The AGE concentrations of each sample were read from the calibration curves for Glu-AGE-BSA, Fru-AGE-BSA, CML-BSA, or Glycer-AGE-BSA standards and were expressed as Glu-AGE, Fru-AGE, CML, or Glycer-AGE units (U) per ml, where 1U corresponded to 1 μg of the Glu-AGE-BSA, Fru-AGE-BSA, CML-BSA, or Glycer-AGE-BSA standard [13–16].

### Assessment of the concentrations of various AGEs

Commonly consumed beverages and foods were obtained from vending machines, convenience stores, supermarkets, fast food stores (including doughnut or hamburger stores), bento-ya (shops that sell lunch sets), or family restaurants. Samples of beverages and liquefied foods (mainly seasonings) were analyzed using competitive ELISA after they had been diluted. To prepare food samples for the AGE measurements, solid food was first crushed uniformly with a food processor (Cuisinart, Mini-Prep Processor/Little Pro Plus, Tokyo, Japan). In the case of the bento-ya/convenience store lunch boxes, any fish, meat, vegetables (including nimono/aemono), seasonings and spices, and rice within them were examined separately, whereas for hamburgers, any meat/fish, vegetables, sauce, and bread/rice were examined separately. We then weighed out 5 g of the uniformly crushed food, added 45 ml of the sample dilution buffer (Tris/HCl buffer containing 0.05% Tween-20, pH 7.4), and homogenized it for 1 min at 15,000 rpm using an Ace Homogenizer (Nippon Seiki Co., Ltd., Nagaoka, Japan). The homogenate was rotated with a tube rotator (AS ONE, AM-9, Osaka, Japan) for 3 h at room temperature. After being centrifuged for 20 min at 3,500 rpm at 4°C, the supernatant was used for the assessments of AGE levels. We purchased at least 2 of each beverage and food, and prepared at least 2 samples of each product for AGE measurement. The concentrations of the four abovementioned types of AGEs in each beverage or food extract were measured using competitive ELISA after the extract had been diluted 10- to 100,000-fold (after controlling for dilution) with sample dilution buffer. The AGE concentrations of each beverage and food item are shown as mean values of at least three measurements per sample and are expressed as AGE units (U). The tests for the four kinds of AGEs displayed sensitivity values of 0.1 U/ml. In the case of the bento-ya/convenience store lunch boxes, the AGE concentrations of any fish, meat, vegetables (including nimono/aemono), seasonings and spices, and rice were combined and expressed as mean units per meal/lunch box. For hamburgers, the AGE concentrations of any meat/fish, vegetables, sauce, and bread/rice were combined and expressed as mean units per hamburger. The concentrations of the four kinds of AGEs were calculated based on standard serving sizes (65~500 ml/bottle) for beverages or on the standard serving sizes consumed by average Japanese people for foods (e.g., one bag/box of confectionery, one bag/box of prepared foods, 15 (~150 for noodle tsuyu) g/ml seasonings and spices, one hamburger, and one convenience store lunch box).

## Results

### Assessment of the AGE concentrations of common beverages as determined by their AGE levels

**Fig 2:**
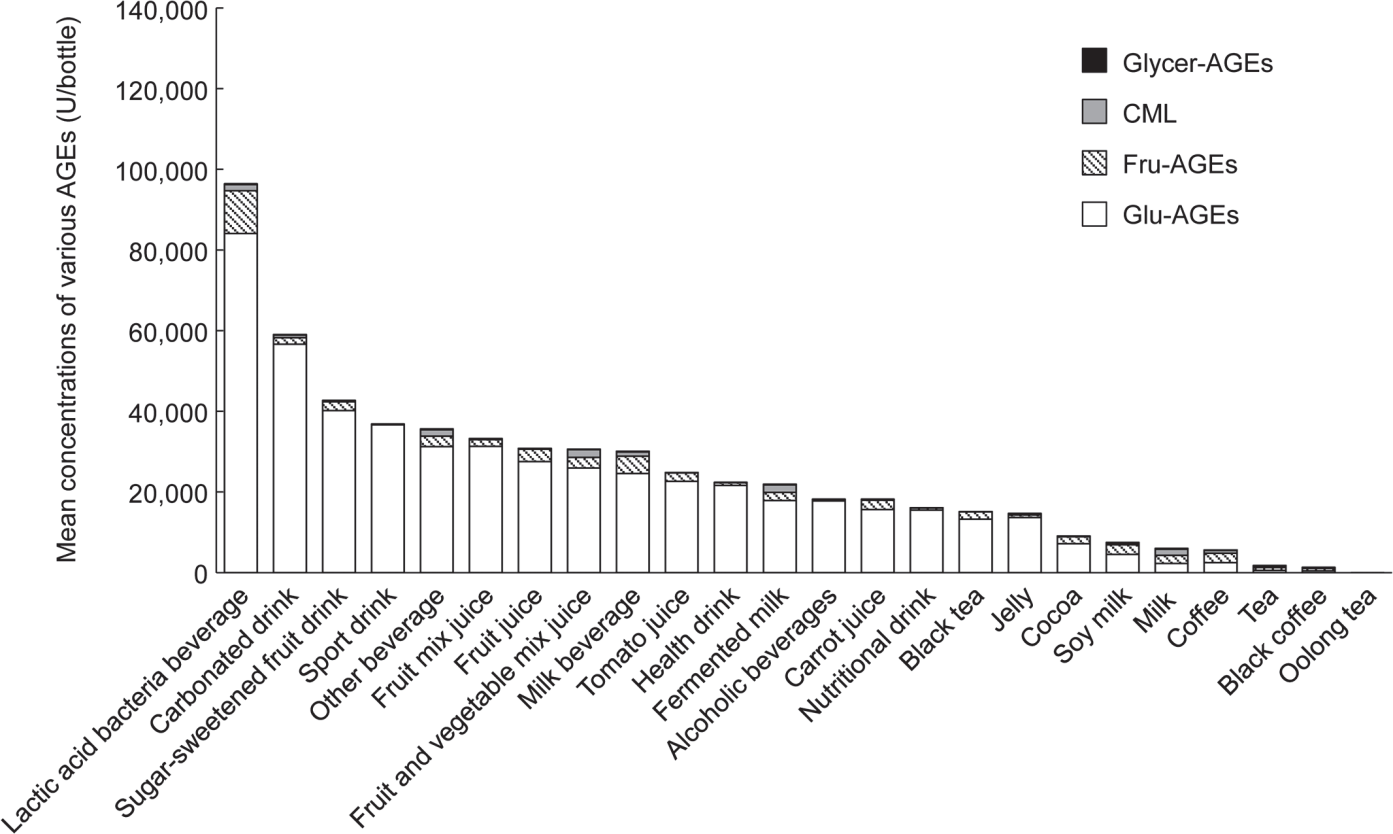
Mean concentrations of various AGEs in commonly consumed beverages. Beverages were classified according to the Japanese Agricultural Standard (JAS). The mean concentrations of four kinds of AGEs (Glu-AGEs, Fru-AGEs, CML, and Glycer-AGEs) in each beverage are expressed as AGE units (U) per bottle and are based on the standard serving size (65~500 ml/bottle) of each product in Japan.

We classified beverages according to the Japanese Agricultural Standard (JAS). The concentrations of the four kinds of AGEs in each type of beverage are shown in Fig. 2. Glu-AGEs, Fru-AGEs, CML, and Glycer-AGEs exhibited concentrations of ≥85%, 2–12%, <3%, and trace amounts, respectively. Table 1 shows a list of commonly consumed beverages that exhibited Glu-AGE concentrations of ≥100,000 U/bottle. The highest Glu-AGE concentrations were detected in the lactic acid bacteria beverages Pil∙Cres (two types; 264,090 and 240,870 U/65 ml bottle), Yakult (eight types; max: 243,890—min: 60,900 U/65 or 80 ml bottle), and lactic acid bacteria Power Peach (171,320 U/250 ml bottle). The lactic acid bacteria beverages containing large amounts of Glu-AGEs were all produced *via* processes in which high-fructose corn syrup (HFCS) and skimmed milk were mixed and reacted at high temperature, which would have altered the mixture’s Glu-AGE levels, before the seeding and culturing of lactic acid bacteria. On the other hand, the lactic acid bacteria beverages that had been colored using caramel had low levels of Glu-AGEs. Moreover, beverages that contained artificial sweeteners and carbonated drinks displayed low levels of Glu-AGEs. Tea, black coffee, and oolong tea did not contain many Glu-AGEs. The numbers of beverages in each category that contained ≥100,000; 50,000–99,999; 20,000–49,999; and <20,000 U/bottle of Glu-AGEs are shown in Table 2. The concentration of Glu-AGEs was ≥20,000 U/bottle in *ca*. 45% of the beverages examined.

**Table 1:** List of common beverages containing Glu-AGE levels of ≥100,000 U/bottle.

Name	AGE concentrations (U/bottle)*				Serving size(ml)
	Glu-AGEs	Fru-AGEs	CML	Total AGEs	
Pil∙Cre Probiotics	264,090	32,750	140	296,980	65
Pil∙Cre Sawayakajitate	240,870	24,450	130	265,450	65
Yakult	243,890	18,570	140	262,600	65
Fanta Fruit Punch	232,540	1,290	330	234,160	500
Natchan! Soda (Junnsuijitate Fruity Grape)	201,810	1,350	0	203,160	500
Fanta Grape	200,250	1,990	0	202,240	500
Yakult 300V LT	164,150	17,410	180	181,740	80
Prune 100	149,870	29,140	1,190	180,200	125
Lactic Acid Bacteria Power Peach	171,320	4,600	320	176,240	250
Canada Dry Ginger Ale Extra	173,130	1,930	0	175,060	500
Yakult 300V	145,940	17,250	170	163,360	80
Blue Ginger	151,350	2,120	480	153,950	500
Fanta Orange	146,830	850	130	147,810	500
Yakult SHEs	123,270	19,970	190	143,430	80
Yakult 400	124,990	14,050	140	139,180	80
Mountain Dew	130,310	2,400	90	132,800	500
Real KIAIDA Kiaida-!!	126,880	1,330	130	128,340	280
Lemons Lemon	121,050	5,670	850	127,570	140
Yakult 80 Ace	110,380	15,730	210	126,320	80
Fanta Green Apple	119,490	1,290	120	120,900	500
Lipton Sparkling Tea Soda (Cassis & White grape)	119,300	410	70	119,780	500
Mitsuya Cider (Sukattoshiroi)	100,470	12,920	3,420	116,810	500
Orange (TOPVALU)	108,320	5,210	1,120	114,650	500
Kokoichibann! Macano-gennki	104,460	3,150	30	107,640	100
Tottemo Orange (Qoo)	103,930	2,590	240	106,760	500
ORANGE ADE (Kobe-kyoryuuchi)	105,040	1,680	40	106,760	350
Junnsuimikann (Koiwai)	100,800	3,190	190	104,180	500

*the Glycer-AGE concentrations of these products are not shown because they were too low to assess accurately.

**Table 2:** The number of beverages that had their Glu-AGE concentrations tested (n = 885).

	(U/bottle)				
	Mean Glu-AGE concentrations	≥100,000	50,000–99,999	20,000–49,999	<20,000
Beverages (660):					
Carbonated Drinks (70)	56,670	12	19	21	18
Oolong Tea (7)	0				7
Black Tea (24)	13,240		1	7	16
Fruit Juice (184):					
Fruit Juice (64)	27,530	1	7	26	30
Mixed Fruit Juice (27)	31,350		4	12	11
Mixed Fruit and Vegetable Juice (28)	25,900		1	16	11
Sugar-sweetened Fruit Drinks (65)	40,200	4	16	21	24
Tomato Juice (16)	22,660			11	5
Carrot Juice (5)	15,620			2	3
Coffee (65):					
Black Coffee (23)	470				23
Coffee (42)	2,500			2	40
Soy Milk (22)	4,520			2	20
Other Beverages (267):					
Sports Drinks (14)	36,670		4	7	3
Health Drinks (13)	21,640		2	3	8
Nutritional Drinks (35)	15,540		1	10	24
Cocoa (10)	7,190		1		9
Jelly (53)	13,630			19	34
Tea (36)	570				36
Other Beverages (106)	31,260	1	20	45	40
Milk and Dairy Products (90):					
Milk (11)	2,270				11
Dairy Products (79):					
Fermented Milk (18)	17,880			6	12
Milk-based Beverages (35)	24,570		6	12	17
Lactic Acid Bacteria-containing Beverages (26)	84,100	9	13		4
Alcoholic Beverages (135)	17,750		6	50	79
**(Number of beverages)**		**(27)**	**(101)**	**(272)**	**(485)**

### Assessment of the AGE concentrations of common foods as determined by their AGE levels

**Fig 3:**
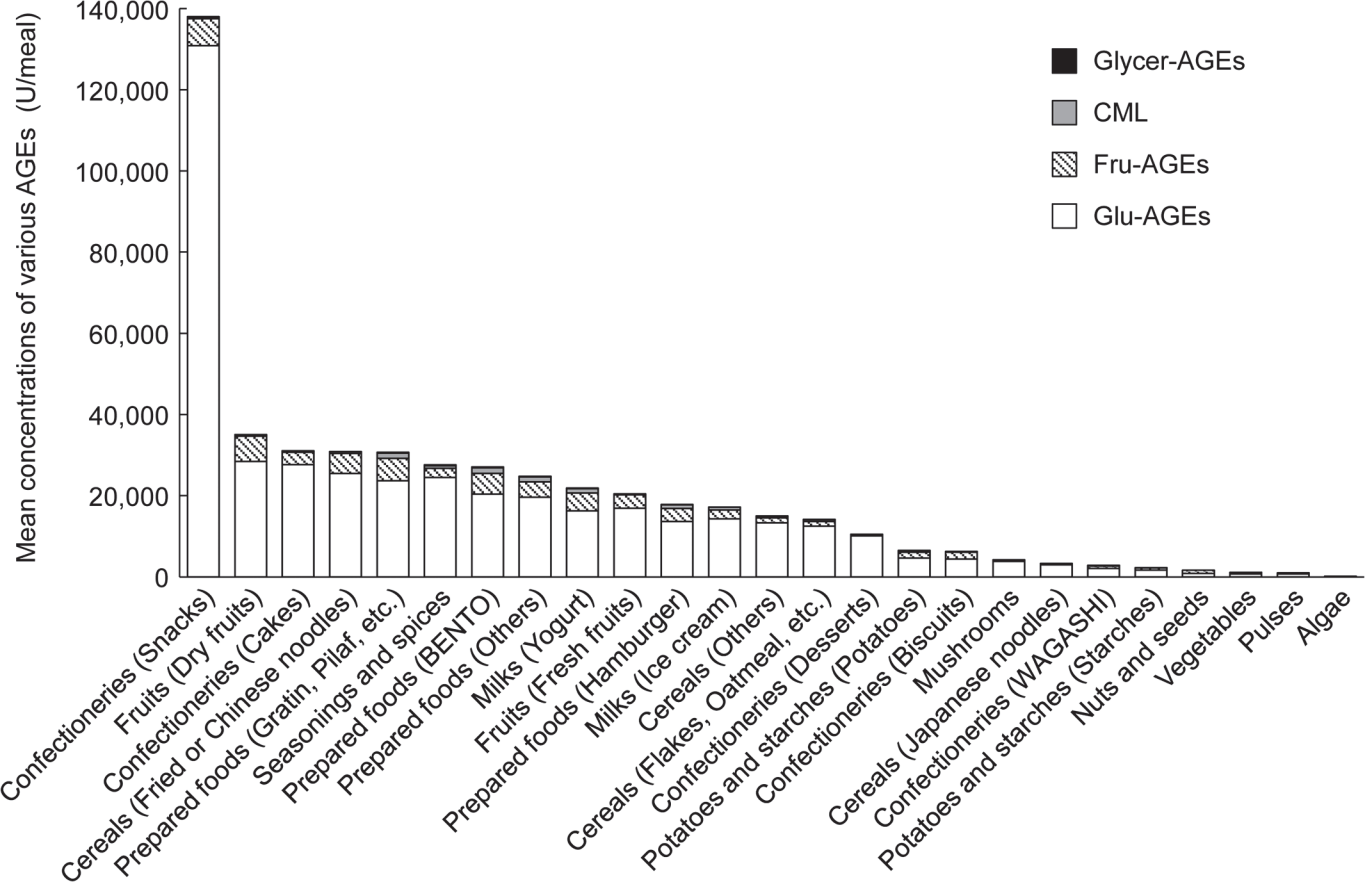
Mean concentrations of various AGEs in commonly consumed foods. Food items were classified according to the Standard Tables of Food Composition (5th revised and enlarged edition, 2009). The mean concentrations of four kinds of AGEs (Glu-AGEs, Fru-AGEs, CML, and Glycer-AGEs) in each food item are expressed as AGE units (U) per meal and are based on the standard servings consumed by average individuals in Japan.

We classified the foods according to the Standard Tables of Food Composition in Japan (5th revised and enlarged edition, 2009). The concentrations of the four kinds of AGEs were measured in over 750 commercially available foods based on the standard servings consumed by average Japanese people. The concentrations of the four kinds of AGEs in each type of food are shown in Fig. 3. Glu-AGEs, Fru-AGEs, CML, and Glycer-AGEs exhibited concentrations of ≥82%, 5–15%, <3%, and trace amounts, respectively. Commonly consumed foods that demonstrated Glu-AGE levels of ≥100,000 U/meal are shown in Table 3. The highest Glu-AGE levels in the small food group were detected in snacks that had been prepared *via* dry-heat processing. These products contained high levels of Glu-AGEs because the processes used to produce them involved the heating of raw materials containing large amounts of reducing sugars (such as HFCS and dried fruit) and soybean flour or flour (which contain large quantities of lysine) at a high temperature for a long period of time. On the other hand, prepared foods such as bentos containing many vegetables and broiled fish contained low levels of Glu-AGEs. Moreover, the algae, vegetables, and pulses did not contain high levels of Glu-AGEs. The numbers of foods in each category that contained ≥100,000; 50,000–99,999; 20,000–49,999; and <20,000 U/meal of Glu-AGEs are shown in Table 4. Approximately 23% of the foods examined contained ≥20,000 U of Glu-AGEs per meal.

**Table 3:** List of common foods with Glu-AGE levels of ≥100,000 U/meal.

Name	AGE concentrations (U/meal)*				Serving size (g)
	Glu-AGEs	Fru-AGEs	CML	Total AGEs	
SOYJOY Apricot	893,780	25,340	500	919,620	30
SOYJOY Mango + Coconut	834,100	22,100	500	856,700	30
SOYJOY Raisin + Almond	828,010	21,450	530	849,990	30
SOYJOY Hawthorn	788,720	20,200	450	809,370	30
SOYJOY Prune	540,530	14,110	520	555,160	30
SOYJOY Strawberry	506,520	25,090	520	532,130	30
SOYJOY Cocoa +Orange	456,650	23,050	520	480,220	30
SOYJOY Apple	453,590	21,280	520	475,390	30
Light Meal Soy Bar Raisin + Almond Flavor	433,320	19,190	480	452,990	27
Rice au Gratin (Joutou-youshoku)	335,710	15,360	2,340	353,410	250
Light Meal Block Fruit Flavor	282,780	37,060	600	320,440	41
Light Meal Soy Bar Pineapple Flavor	277,760	12,210	470	290,440	27
SOYJOY Blueberry	270,210	18,500	460	289,170	30
Old Fashion	250,770	3,900	760	255,430	62
Light Meal Soy Bar Cocoa + Orange Flavor	206,450	11,570	420	218,440	27
Eel Bento	203,340	7,540	4,430	215,310	(a meal)
Chocolate Fashion	206,990	3,180	460	210,630	65
Light Meal Block Blueberry Yogurt Flavor	192,430	12,940	610	205,980	41
Calorie Mate Block Fruit Flavor	178,720	10,310	270	189,300	40
Old Fashion Maccha Green Tea	179,710	2,890	430	183,030	62
SOYJOY Banana	174,920	4,990	120	180,030	30
Old Fashion Maccha Green Tea Chocolate	161,010	4,410	400	165,820	64
SOYJOY Orange	157,750	5,770	550	164,070	30
Hamburger Steak with Sauce (Ogawaken)	145,610	10,430	5,040	161,080	210
Natsukashino Sauce Yakisoba	144,150	8,660	440	153,250	184
Tomato Ketchup (Kagome)	150,040	3,040	130	153,210	15
Pudding Super BIG (Meiji)	137,340	12,120	250	149,710	200
Hayashi Rice (Ogawaken)	135,400	7,500	1,150	144,050	350
Organic Tomato Ketchup (Del Monte)	140,710	2,220	80	143,010	15
Haagen-Dazs Bitter Caramel	126,210	3,990	90	130,290	120
Honnkaku Okonomi Sauce (Otafuku)	125,290	1,580	150	127,020	15
Ippei-chan Yomiseno Yakisoba	115,920	4,920	1,210	122,050	265
Hiyashichuuka Soup Soy Sauce	101,590	7,670	2,580	111,840	150
GYU-DON (Kinno-donburi)	104,790	2,660	1,500	108,950	180
HAYASHI (Curry-ya)	103,260	4,540	400	108,200	210

*the Glycer-AGE concentrations of these products are not shown because they were too low to assess accurately.

**Table 4:** The number of food items that had their Glu-AGE concentrations tested (n = 767).

	(U/meal)				
	Mean Glu-AGE concentrations	≥100,000	50,000–99,999	20,000–49,999	<20,000
Cereals (87):					
Fried or Chinese Noodles (22)	25,510	2	3	3	14
Flakes, Oatmeal, and Rice (10)	12,520		1	2	7
Japanese Noodles (14)	2,970			1	13
Others (41)	13,300		3	6	32
Potatoes and Starches (27):					
Potatoes (8)	4,550				8
Starches (19)	1,680				19
Pulses (15)	770				15
Nuts and Seeds (8)	900				8
Vegetables (39)	740				39
Fruits (29):					
Fresh Fruit (21)	16,910			7	14
Dried Fruit (8)	28,400		1	2	5
Mushrooms (12)	3,800				12
Algae (22)	160				22
Milks (44):					
Yogurt (16)	16,310		3	1	12
Ice Cream (28)	14,300	1		5	22
Confectionery (197):					
Snacks (60)	130,840	17	1	4	38
Cakes (49)	28,060	4	1	6	38
Desserts (45)	10,110	1		1	43
Biscuits (27)	4,420			1	26
Wagashi (16)	2,080				16
Seasonings and Spices (78)	24,540	4	4	24	46
Prepared Foods (209):					
Gratin, Pilaf, or Fried Rice (27)	23,650	1	1	3	22
Bento (48)	20,430	1	1	16	30
Hamburger (41)	13,670			11	30
Others (93)	19,630	4	5	21	63
**(Number of food items)**		**(35)**	**(24)**	**(114)**	**(594)**

## Discussion

A previous study indicated that a significant proportion of pro-inflammatory AGEs are derived from dietary components [28]; however, it is disputed whether such AGEs are a health risk [8,28–30]. ELISA or liquid chromatography-mass spectrometry (LC-MS) are usually used to assess the levels of AGEs in bodily secretions, foods, and beverages. However, it is not possible to assess the levels of both HMW- and LMW-AGEs using any of the current approaches. Subjecting foods to heating results in the formation of various AGE molecules, with the types of AGEs produced depending on the heat treatment method employed and the food involved. Unfortunately, most previous studies only examined the levels of a small number of AGE molecules because of methodological issues [31,32]. The majority of studies of AGEs have focused on representative molecules, particularly CML. Based on assessments of CML levels obtained with ELISA, a database of food products and their AGE concentrations has been produced [6,7]. Foods containing elevated levels of protein and fat, meat substitutes, and processed meats were found to display markedly increased AGE concentrations. However, discrepancies have been detected between the information in the abovementioned database and the data obtained using other approaches, which indicates that more accurate methods for analyzing AGE levels are required [33]. As AGEs are produced when food is heated, eating processed foods, which are subjected to high temperatures during their production, results in greater exposure to AGEs. The Maillard reaction, in which sugar groups react with proteins (which causes foods to turn brown) and cross-links form between proteins, is responsible for AGE synthesis during the heating of foodstuffs [31]. Conversely, CML is colorless and does not promote protein cross-linking or fluoresce [2–5].

There are no established methods for evaluating the serum concentrations of AGEs. Whilst most studies of AGEs have examined CML levels, the role played by CML in pathological conditions is poorly understood. Although numerous other AGE structures are known to exist, little is known about which AGEs make significant contributions to disease. In previous studies, we found that the serum concentrations of Glycer-AGEs, but not those of Glu-AGEs, CML, or HbA1c, could be useful biomarkers for predicting the development of cardiovascular events and atherosclerosis [21–25]. Hence, in the present study we subjected a range of foods and beverages that are commonly consumed in Japan to testing in order to determine their AGE concentrations (their CML, Glu-AGE, Glycer-AGE, and Fru-AGE concentrations). The present study indicates that CML is not a suitable biomarker of dietary AGE consumption (Figs. 2 and 3 and Tables 1 and 3). The findings of the present study indicate that people should not consume excessive amounts of particular types of food such as cereals, dried fruits, seasonings, prepared foods, and confectionery (snacks) or certain beverages, particularly sugar-sweetened fruit drinks, lactic acid bacteria beverages, mixed fruit juices, sports drinks, and carbonated drinks.

Interestingly, the present study demonstrated that a markedly greater number of foods and beverages contain Glu-AGEs than Fru-AGEs, even among dried fruit products and fruit juices, which contain large amounts of fructose. During glycation, the initial kinetics of the reaction are affected by the protein involved, the temperature at which the reaction occurs, the concentration of the reducing sugar, and the percentage of the reducing sugar that possesses an open-chain structure. Compared with glucose, a greater proportion of fructose exhibits an open-chain structure. Studies of glycation have found that the initial rates of fructose-adduct formation are increased in Hb; however, glucose and fructose display similar levels of reactivity with RNase A, and glucose reacts with albumin 8 times more readily than fructose. The discrepancies between these findings might be due to differences in the local conditions at the reaction sites [34]. A Japanese study examined the amounts of glucose and fructose in various fruits. As a result, it was found that the levels of glucose and fructose in 100 g of fruit were as follows: 1.5 g and 1.5 g, respectively, in oranges; 3.0 g and 5.5 g, respectively, in apples; 7.5 g and 8.0 g, respectively, in grapes; and 4.0 g and 2.5 g, respectively, in bananas. Therefore, the detection of Glu-AGEs in fruit seems reasonable, although they contain even more Fru-AGEs. Irrespective of the proteins involved, AGEs are defined as molecules that contain AGE structures. It has been demonstrated that Glu-AGE structures often develop in casein that has reacted with glucose as well as in RNase A, Hb, and albumin. In the present study, the IC_50_ values of three types of AGE-BSA or CML-BSA standards were found to range from 1.0–1.5 U/mL in competition experiments involving an anti-CML antibody and three anti-AGE antibodies (data not shown); therefore, it is considered that all three anti-AGE antibodies and the anti-CML antibody had similar ability to recognize AGEs in beverages and foods.

When assessing the levels of AGEs using ELISA, it is essential to be aware of the cross-reactivity of the antibodies employed. Detailed assessments of the relevant epitopes are necessary to ensure this, together with precise knowledge of the target structure. In addition, it is necessary to validate the ELISA in each matrix, e.g., using spiking, as the local chemical milieu can affect antigen-antibody binding [8]. At present, our knowledge of the various complex pathways involved in AGE synthesis is insufficient. As AGE synthesis pathways are complex and exhibit a great degree of diversity, a large number of molecules are defined as AGEs. A significant number of dietary AGEs, including argpyrimidine, CEL, pentosidine, imidazolones, CML, pyrraline, GOLD, DOLD, and MOLD, *etc*., have been detected *in vivo* [35,36]. In previous studies, we have found that anti-Fru-AGE, anti-Glycer-AGE, and anti-Glu-AGE antibodies recognize epitopes that differ from previously described AGE structures [13–16,23,27]; i.e., it is suggested that these AGE antibodies recognize novel AGE structures.

In humans, it has been demonstrated that roughly 10% of the AGEs in foods and beverages are taken up into the body. Of these, ~33% are excreted in urine within 48 h of their consumption, while ~67% accumulate within the body [37]. In a study conducted in the USA, Semba *et al*. stated that adults exhibit median (25^th^/75^th^ percentile) serum CML concentrations of 0.69 (0.60/0.80) μg/ml [11]. In addition, non-diabetic individuals with normal renal function were found to have a mean serum Glu-AGE level of 10.5±1.3 U (one unit equals 1 μg of Glu-AGE-BSA)/ml, whereas diabetic patients and diabetic patients on hemodialysis exhibited serum Glu-AGE concentrations that were >2 times greater (24.7±2.4 U/ml) and ~8 times greater (79.4±9.9 U/ml), respectively, than those seen in the non-diabetic subjects [38]. We examined the serum Glu-AGE levels of healthy subjects and Japanese diabetic nephropathy patients and found that they were 10–20 U/ml and 30–50 U/ml, respectively. The consumption of 100,000 U of dietary Glu-AGEs results in a blood Glu-AGE level of ~2.0 U/ml [100,000 (U) × 0.1 (the proportion that is absorbed) x 1/5,000 (ml of blood)]. The dietary intake of food products containing <20,000 U Glu-AGEs has little effect on the body. On the other hand, care should be taken when mixing Glu-AGE-containing products or consuming large amounts of Glu-AGE-containing beverages or foods as this can result in elevated concentrations of Glu-AGEs and sugar in the blood and promote the hepatic build-up of Glu-AGEs [20,26].

In a previous study, we detected elevated hepatic RAGE expression in normal rats that had been given a Glu-AGE-rich beverage (Yakult) [20]. In addition, the rats’ hepatic cells were found to contain Glycer-AGEs and Glu-AGEs, despite the fact that the beverage administered to the rats did not contain Glycer-AGEs. The above findings suggest that the synthesis and hepatic accumulation of Glycer-AGEs are promoted by Glu-AGEs, which are often found in foods and beverages, resulting in increased Glycer-AGE-RAGE binding [20,26]. In another study, AST-120 (Kremezin, Kureha-Chemical Co., Tokyo, Japan), an oral adsorbent that slows the development of chronic renal failure (CRF) by promoting the removal of uremic toxins, reduced the serum Glu-AGE and Glycer-AGE concentrations of non-diabetic CRF patients [26]. In addition, in an examination of the expression profiles of endothelial cells extracted from the latter patients’ serum samples the mRNA expression levels of monocyte chemoattractant protein-1, vascular cell adhesion molecule-1, and RAGE were found to be significantly downregulated in the cells acquired after AST-120 (Kremezin) treatment compared with those seen in the endothelial cells obtained prior to treatment [26]. The latter results indicated that the consumption of Glu-AGEs might contribute to the development of vascular damage in pathological conditions linked to Glycer-AGE-RAGE interactions. Furthermore, they suggest that reducing the absorption of dietary Glu-AGEs might be a useful strategy for treating lifestyle-related conditions. Further clinical studies might help to elucidate whether lifestyle-related conditions can be prevented by encouraging people to reduce their consumption of Glu-AGEs.

In conclusion, we have presented useful information regarding the AGE concentrations of numerous beverages and foods that are commonly consumed in Japan. As dietary AGEs are derived from numerous precursors, they include a broad range of compounds with different structures and molecular weights. There is insufficient detailed data about the functions and molecular structures of AGEs. Furthermore, little is known about the *in vivo* activity of AGEs or about their bioavailability and absorption. This is partly because of a dearth of accurate analytical techniques for assessing the concentrations of AGEs in food and tissues. The structures of the epitopes recognized by anti-Fru-AGE, anti-Glycer-AGE, and anti-Glu-AGE antibodies were not examined in the present study; however, we were able to determine that they differ from those of well-defined AGEs as well as those of AGEs derived from carbonyl or sugar molecules with unknown structures. Accordingly, it is possible that Glycer-AGEs, Fru-AGEs, and Glu-AGEs have unique structures, but studies involving spectroscopic and biochemical analyses are required to confirm this.
